# Intake of Farmed Atlantic Salmon Fed Soybean Oil Increases Insulin Resistance and Hepatic Lipid Accumulation in Mice

**DOI:** 10.1371/journal.pone.0053094

**Published:** 2013-01-02

**Authors:** Lisa Kolden Midtbø, Mohammad Madani Ibrahim, Lene Secher Myrmel, Ulrike Liisberg Aune, Anita Røyneberg Alvheim, Nina S. Liland, Bente E. Torstensen, Grethe Rosenlund, Bjørn Liaset, Trond Brattelid, Karsten Kristiansen, Lise Madsen

**Affiliations:** 1 Department of Biology, University of Copenhagen, Copenhagen, Denmark; 2 National Institute of Nutrition and Seafood Research, Bergen, Norway; 3 Institute of Biomedicine, University of Bergen, Bergen, NorwayStavanger, Norway; Univeristy of California Riverside, United States of America

## Abstract

**Background:**

To ensure sustainable aquaculture, fish derived raw materials are replaced by vegetable ingredients. Fatty acid composition and contaminant status of farmed Atlantic salmon (*Salmo salar L.*) are affected by the use of plant ingredients and a spillover effect on consumers is thus expected. Here we aimed to compare the effects of intake of Atlantic salmon fed fish oil (FO) with intake of Atlantic salmon fed a high proportion of vegetable oils (VOs) on development of insulin resistance and obesity in mice.

**Methodology/principal findings:**

Atlantic salmon were fed diets where FO was partly (80%) replaced with three different VOs; rapeseed oil (RO), olive oil (OO) or soy bean oil (SO). Fillets from Atlantic salmon were subsequently used to prepare Western diets (WD) for a mouse feeding trial. Partial replacement of FO with VOs reduced the levels of polychlorinated biphenyls (PCB) and dichloro-diphenyl-tricloroethanes (DDT) with more than 50% in salmon fillets, in WDs containing the fillets, and in white adipose tissue from mice consuming the WDs. Replacement with VOs, SO in particular, lowered the n−3 polyunsaturated fatty acid (PUFA) content and increased n−6 PUFA levels in the salmon fillets, in the prepared WDs, and in red blood cells collected from mice consuming the WDs. Replacing FO with VO did not influence obesity development in the mice, but replacement of FO with RO improved glucose tolerance. Compared with WD-FO fed mice, feeding mice WD-SO containing lower PCB and DDT levels but high levels of linoleic acid (LA), exaggerated insulin resistance and increased accumulation of fat in the liver.

**Conclusion/Significance:**

Replacement of FO with VOs in aqua feed for farmed salmon had markedly different spillover effects on metabolism in mice. Our results suggest that the content of LA in VOs may be a matter of concern that warrants further investigation.

## Introduction

The ability of marine n−3 polyunsaturated fatty acids (PUFAs) to protect against the development of cardiovascular disease is well documented [1]–[3]. Thus, increasing the dietary intake of n−3 PUFAs is currently recommended by several health authorities. During the recent years, there has been increasing focus on the possible ability of n−3 PUFAs to protect against other life-style diseases such as obesity and type 2 diabetes. Although human studies are inconclusive [4], rodent feeding trials demonstrate that marine n−3 PUFAs are able to protect against development of obesity [5]–[10], and insulin resistance [9], [11]–[15]. Fish and seafood, in particular fatty fish such as Atlantic salmon (*Salmo salar* L.), are rich in n−3 PUFAs. Thus, both health authorities and consumers have accepted farmed Atlantic salmon as part of a healthy diet. Farmed Atlantic salmon have traditionally been fed diets with high levels of fish meal and fish oil (FO), and a single meal of 200 g Atlantic salmon, providing 4 g of n−3 PUFAs, would be sufficient to cover more than the weekly recommended intake of n−3 PUFAs [16]. However, due to the pressure on wild fish stocks, and hence limited access and variable prices of fish meal and FO for the rapidly growing global aquaculture industry [17], efforts are channeled towards development of aqua feed that rely less on fish meal and FO [18], [19].

Vegetable oils (VOs) are presently recognized as suitable alternatives in aqua feed [20], and consequently, the nutrient composition in the Atlantic salmon fillets will change [16]. Although the Atlantic salmon have the ability to elongate and desaturate fatty acids [21], [22], Atlantic salmon fed vegetable feed have lower content of marine n−3 PUFAs [16], [22] and a direct relationship has been reported between marine n−3 PUFA content in aqua feed, Atlantic salmon fillets and plasma in patients with coronary heart disease (CHD) consuming the fillets [23]. Documentation related to the health beneficial effects of fish has almost exclusively been based on trials using FO, and therefore focused on the content of marine n−3 PUFAs in seafood. Thus, an important question is whether the health beneficial effects are preserved when the Atlantic salmon are fed VOs at the expense of FO. A recent study by Alvheim et al. has demonstrated that an increased content of linoleic acid (LA) from soybean oil (SO) in salmon feed increased the endocannabinoid tone, both in the Atlantic salmon and in the mice fed diets containing the salmon fillets [24]. The increased endocannabinoid tone in the mice was accompanied by increased weight gain.

Persistent organic pollutants (POPs) accumulate in lipid-rich food, and thus, Atlantic salmon represent a source of POPs in addition to marine n−3 PUFAs. The levels of POPs in wild fish are determined by factors including fat content, prey and geographic location (Seafood data, www.nifes.no), whereas contamination in farmed fish mainly depends on the composition of the fish feed [25]. Thus, replacing marine ingredients in aqua feed with vegetable sources will change the composition of nutrients and the type and levels of POPs in the fish fillets [25], [26]. For instance, Atlantic salmon fed higher amounts of vegetable ingredients have lower fillet levels of dioxins and polychloridinated biphenyl (PCB) congeners compared to salmon fed FO [25]. This may be of importance for the consumers as POP exposure has been associated with development of type 2 diabetes in humans [27]–[31]. We have recently demonstrated that intake of purified salmon oil attenuated development of insulin resistance and obesity in Wistar rats, whereas intake of crude salmon oil containing POPs exaggerated obesity development [32]. Moreover, we have recently shown that chronic consumption of Atlantic salmon with a high level of POPs caused insulin resistance and obesity in mice [33]. When the levels of POPs in the salmon were reduced, development of insulin resistance and obesity in the mice decreased concomitantly [33].

Since both the nutrient and contaminant status of Atlantic salmon are affected by the use of plant-based feed ingredients, a spillover effect on consumer health might be expected. In the present study we compared the development of insulin resistance and obesity in mice fed a western type diet containing either Atlantic salmon fed FO or Atlantic salmon where the FO in the feed was replaced by a high proportion of VOs. Our results indicate that the altered fatty acid composition, the increased content of linoleic acid (LA) in particular, rather than the altered content of POPs, in fillets from Atlantic salmon, influenced development of insulin resistance and hepatic accumulation of lipids in mice.

## Materials and Methods

### Ethical Statement

Animal handling and experiments were performed in accordance with the guidelines of the Norwegian State Board of Biological Experiments with Living Animals (Norwegian approval identification nr, FOTS Id: 3196). No adverse events were observed.

### Production of Atlantic Salmon

The salmon feeding trial was carried out at Skretting ARC, Fish Trials Station, Stavanger, Norway. A total of 600 Atlantic salmon (*Salmo salar L.*), mean weight 815±28 g, were randomly distributed in 12 tanks. The salmon were divided into four experimental groups and fed different experimental diets for 28 weeks as described earlier [22]. The fat source in the control diet was 100% FO, whereas 80% of the FO was replaced by rapeseed oil (RO), olive oil (OO), or soy bean oil (SO), in the experimental diets. These oils were chosen based on their different n−6 PUFA content [22]. In all diets 70% of fish meal was replaced with plant proteins. The complete dietary compositions and analyses of the diets and fillets have been reported earlier [22]. Upon sampling, salmon fillets were freeze dried, homogenized and analyzed for fat and protein content.

### Mouse Diets

All diets formulations were based on the standard Western diet (WD), D12079B Research Diets, Inc. NJ, USA prepared by Ssniff Spezialdiäten (Soest, Germany). We replaced 50% of the protein source in the standard Western diet with proteins from salmon fed FO, RO, OO or SO. Since the fat content differed in salmon fed the different diets, milk fat was used to achieve isocaloric diets. The major fatty acid source in the milk fat used was 16∶0, 18∶1, 14∶0 and 18∶0, representing, 29, 26, 10 and 11%, respectively. Composition of the mice diets are shown in Table 1. As references, two groups of mice were fed either a casein based standard WD (Ssniff S8672), or a regular low fat diet (LF), D12450B (Ssniff EF R/M Control, Germany). Complete fatty acid composition of the WD and LF diet is available at http://www.ssniff.com/documents/10_catalogue_ef_engl_1.pdf.

**Table 1:** Composition of the experimental mice diets (g/kg).

	WD-FO	WD-RO	WD-OO	WD-SO	LF	WD
**Components added (g/kg)**						
Casein	97	97	97	97	197	197
Protein from salmon	100	100	100	100	–	–
Milk fat, anhydrous	138	131	129	135	–	200
Fat from salmon	62	69	71	65	–	–
Corn oil	10	10	10	10	70	10
Corn Starch	38	46	41	41	–	50
Maltodextrin	100	100	100	100	539	100
Sucrose	340	340	340	340	90	340
**Analyzed**						
Fat (g/kg)	203	203	195	200	47	194
Crude protein (N*6.25) (g/kg)	171.2	162.9	163.6	163.6	173.1	156.2
Energy kJ/g	21.2	21.1	21.0	21.1	17.1	20.7

All diets were supplemented with 40 mg/kg ethoxyquin, 2g/kg Choline bitartrate, 4g/kg calcium carbonate, 10g/kg Vitamin mix V1001, 35g/kg Mineral mix, 3g/kg L-Methionine and 50g/kg cellulose. Cholesterol levels in WDs were balanced to a final concentration of 1.5 g/kg.

Analyzed values represent the mean of duplicate measurements.

### Animals

48 male C57BL/6J BomTac mice were obtained from Taconic (Ejby, Denmark) at 8 weeks of age. The mice were maintained in a controlled environment with an artificial 12-h-light/dark cycle at thermoneutrality (30°C). Before the start of the feeding experiment animals were allowed to acclimatize to a regular LF diet for 5 d. After acclimatization, the mice were housed individually and randomly assigned to the experimental diets.

The animals were fed the experimental diets described in Table 1 for 10 weeks *ad libitum*. Throughout the experiment, all mice were weighed once a week and feed intake was assessed every Monday, Wednesday and Friday.

### Insulin and Glucose Tolerance Tests (ITT and GTT)

ITT and GTT were performed on conscious mice after 7 and 8 weeks of feeding, respectively. ITT was performed in fed state by intraperitoneal (i.p.) injection of insulin (Actrapid, Novo Nordisk, Bagsværd, Denmark, 0,75 U/kg body weight). GTT was performed in 6 h fasted mice by i.p injection of glucose (2.0 mg/g body weight). Blood was collected from the lateral tail vein and glucose levels were measured at different time-points using a glucometer (Ascensia Contour, Bayer Healthcare, Oslo, Norway). For the GTT, incremental area under the curve (AUC) was calculated as blood glucose concentrations above baseline levels.

### Plasma and Tissue Sampling

At the end of the feeding period, overnight fasted mice were sacrificed by cardiac puncture under Isoflurane anesthesia (Isoba-vet, Schering-Plough, Denmark). Liver, muscles, and adipose tissue depots were quickly dissected out, weighed, snap-frozen in liquid nitrogen and stored at −80°C for further analyses. Blood was collected in tubes containing 10 µl heparin (10 mg/ml, Sigma-Aldrich, United Kingdom), and plasma was prepared by centrifugation. Red blood cells (RBCs) and plasma were stored in aliquots at −80°C for further use.

### Plasma Analyses

A commercial ELISA kit was used in accordance with the manufacturer’s instruction for determination of plasma insulin (Mouse Insulin ultrasensitive ELISA, DRG, Marburg, Germany). Plasma glucose was determined using an automated analyzer (Maxmat PLll, Multi-purpose diagnostic analyzer system, Montpellier, France).

### Lipid Analyses

Total fat content in the diets was measured gravimetrically after acidic hydrolysis and petroleum ether extraction. Tissue lipids were extracted from liver and muscle samples with chloroform: methanol, 2∶1 (v/v). Lipid classes were analyzed using an automated High Performance Thin Layer Chromatography (HPTLC) system (Camaq, Switzerland) and separated on HPTLC plates coated with silica gel 60 F [34]. Fatty acid composition of total lipids in red blood cells was analyzed on a capillary gas chromatograph with flame ionization detector (Perkin Elmer, USA) [35].

### Energy in Feces and Diets

Energy content was determined in a bomb calorimeter following the manufacturer’s instruction (Parr Instruments, Moline, IL, USA).

### Contaminant Analyses

The levels of 7 polychlorinated biphenyls (7 PCBs) and dichloro-diphenyl-tricloroethanes (DDTs) in salmon fillets, mouse feed, liver and adipose tissue from mice were analyzed as described earlier [33]. The 7 PCBs comprise PCB 28, 52, 101, 118, 138, 153 and 180). DDTs comprise op′-DDT, pp′-DDT, op′-DDD, pp′-DDD, op′-DDE and pp′-DDE.

### Statistics

All data are presented as mean ± SEM. Statistical significance was analyzed by using one-way ANOVA, with Dunnett’s Multiple Comparisons test as post hoc test. All groups were compared against the group receiving WD containing salmon fed FO. To compare plasma glucose concentrations during the GTT and ITT over time and between dietary treatments, a 2-way repeated-measures ANOVA was applied. Statistical analysis was performed using Graph Pad Prism version 5.04 (GraphPad Software Inc, La Jolla, CA, USA). *, ** and ***represents significant different from WD containing salmon fed FO at P<0.05, P<0.01 and P<0.001 levels, respectively.

## Results

### Replacing Fish Oil with Vegetable Oils in Fish Feed Reduces the n−3/n−6 Ratio in Atlantic Salmon Fillets and in the Red Blood Cells from Mice Consuming the Salmon Fillets

It is well known that replacing FO with VO in fish feed changes the composition of both nutrients and POPs in Atlantic salmon fillets [22], [25], but the spillover effects of such changes for the consumers are not thoroughly examined. To investigate this, Atlantic salmon were fed diets in which 70% of marine proteins were exchanged with vegetable proteins and 80% of the FO were exchanged with VO. The VOs used were rape seed oil (RO), olive oil (OO) and soybean oil (SO), chosen based on their different content of n−6 PUFAs [22]. Compositions of the salmon diets are listed in Liland et al. 2012 [22]. To investigate the potential spillover implications of such changes in the aqua feed for consumers, we prepared standard WDs containing these salmon filets, replacing 50% of the protein source (casein) with protein from salmon (Table 1). The fat content was slightly different in salmons fed the different feeds, and milk fat, used as fat source in the standard WD diet, was used to achieve isocaloric diets (Table 1). To verify the expected differences in fatty acid composition in the mouse diets, hereafter referred to as WD-FO, WD-RO, WD-OO, and WD-SO, analyses of the diets were performed (Table 2). The diets containing salmon fed either of the VOs, hereafter collectively referred to as WD-VOs, had significantly lower, but comparable levels of the marine n−3 PUFAs, EPA and DHA, than WD-FO (Table 2). Compared with WD-FO, the levels of n−6 PUFAs were higher in WD-VOs (Table 2). Of note, the levels of LA were comparable in WD-RO and WD-OO, but particularly high in WD-SO (Table 2). Furthermore, the levels of the vegetable n−3 PUFA, α-linolenic acid (ALA) was higher in the WD-RO than the other WD-VOs (Table 2). Thus, the n−3/n−6 ratio varied between the WD-VOs. The n−3/n−6 ratio was lower in WD-OO than in WD-RO, and the lowest n−3/n−6 ratio was measured in the WD-SO (Table 2).

**Table 2:** Fatty acid composition of the experimental mice diets.

	WD-FO	WD-RO	WD-OO	WD-SO	LF	WD
Sum SFA (mg/g)	93.2	78.8	84.2	85.2	7.1	103.5
Sum MUFA (mg/g)	58.2	67.1	73.4	55.4	7.6	50.8
LA, 18∶2*n*−6 (mg/g)	9.6	15.6	15.3	25.8	8.0	8.1
AA, 20∶4*n*−6 (mg/g)	<0.1	0.3	0.3	<0.1	<0.1	<0.1
Sum *n*−6 (mg/g)	10.5	17.3	17.0	28.5	8.1	8.3
ALA, 18∶3*n*−3 (mg/g)	1.8	3.9	2.7	2.7	1.0	1.0
EPA, 20∶5*n*−3 (mg/g)	4.1	1.3	1.2	1.2	<0.1	0.1
DHA, 22∶6*n*−3 (mg/g)	6.2	2.6	2.5	2.3	<0.1	<0.1
Sum *n*−3 (mg/g)	18.4	10.9	9.2	9.2	1.1	2.5
n−3/n−6 ratio	1.75	0.63	0.54	0.32	0.13	0.29

Data represent mean of duplicate measurements. Sum n−3 and sum n−6 include additional fatty acids not indicated in the table.

Abbreviations: ALA, α-linolenic acid; AA, arachidonic acid; DHA, docosahexaenoic acid; EPA, eicosapentaenoic acid; LA, linoleic acid, MUFA, monounsaturated fatty acids; SFA, saturated fatty acids.

**Figure 1:**
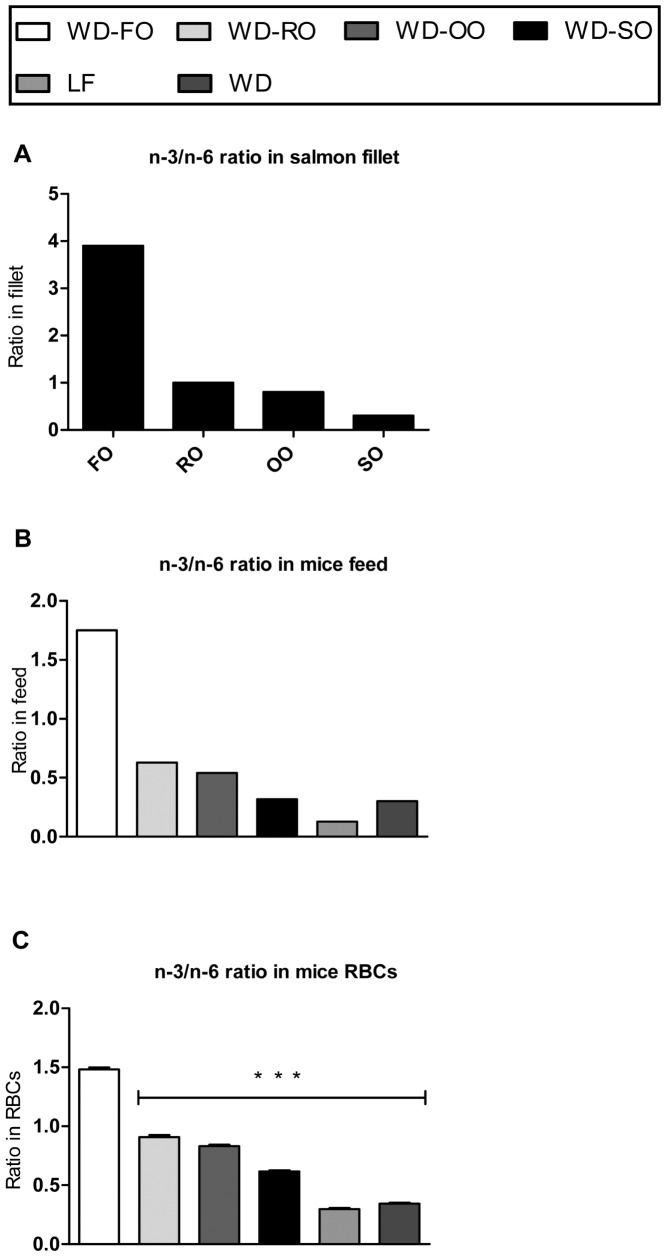
Composition of salmon aqua feed changes n−**3/n**−6 PUFA ratio in mice consuming the salmon fillets. Fish oil (FO) in aqua feed was partly replaced with rapeseed oil (RO), olive oil (OO) or soy bean oil (SO) and fed Atlantic salmon. The salmon fillets were used in Western diets (WDs) fed male C57BL/6J mice (n = 8/diet) for 10 weeks. Fatty acid composition was measured and the n−3/n−6 PUFA ratio calculated in (A) Atlantic salmon fillets (B) the WDs containing the fillets and (C) red blood cells (RBC) collected from mice consuming the WDs and reference diets. Data represent mean of duplicate measurements in A and B and mean+SEM (n = 5) in C. Asterisk(s) indicates significant different from FO-WD.

To investigate possible spillover effects for consumers, C57BL/6J mice were fed these diets for 10 weeks and fatty acid composition was measured in the red blood cells (RBC). As references, two groups of mice were fed either a standard casein based WD or a regular low fat (LF) diet. As predicted, the combined levels of n−6 PUFAs were higher and the levels of n−3 PUFAs lower in RBCs in mice fed WD-VOs compared with WD-FO (Table 3). Thus, the n−3/n−6 ratio in RBCs in the mice reflected the ratio in the salmon fillets and mouse diets (Fig. 1). Compared with the WD-FO diet all the WD-VOs significantly reduced the omega-3 index - with over 30% reduction in mice fed the WD-SO diet compared to mice fed the WD-FO diet (Table 3). The omega-3 indices were reduced 18 and 21% in WD-RO and WD-OO fed mice, respectively (Table 3). However, all mice fed diets containing salmon had an omega-3 index higher than 8% [2], indicating that intake of the WD-VO diets still might exert a cardio-protective effect. For comparison, the reference diets resulted in an omega-3 index below the recommended limit of 8%.

**Table 3:** Fatty acid composition in RBCs from mice after consuming the experimental diets for 10 weeks.

	WD-FO	WD-RO	WD-OO	WD-SO	LF	WD
Sum FA (mg/g)	3.1±0.2	3.1±0.2	3.2±0.1	3.2±0.2	3.1±0.1	3.1±0.1
Sum SFA (mg/g)	1.26±0.06	1.14±0.01*	1.18±0.02	1.19±0.01	1.18±0.01	1.13±0.02*
Sum MUFA (mg/g)	0.58±0.03	0.59±0.01	0.66±0.01*	0.51±0.02	0.61±0.02	0.70±0.02***
LA, 18∶2n−6 (mg/g)	0.23±0.02	0.32±0.01***	0.32±0.02**	0.37±0.01***	0.29±0.02*	0.26±0.01
AA, 20∶4n−6 (mg/g)	0.22±0.01	0.25±0.01	0.27±0.01**	0.32±0.01***	0.56±0.02***	0.51±0.01***
Sum n−6 (mg/g)	0.49±0.03	0.65±0.02***	0.69±0.01***	0.82±0.02***	0.97±0.01***	0.91±0.02***
EPA, 20∶5n−3 (mg/g)	0.21±0.01	0.15±0.01***	0.13±0.01***	0.09±0.01***	0.01±0.00***	0.02±0.00***
DHA, 22∶6n−3 (mg/g)	0.40±0.02	0.34±0.00**	0.35±0.00*	0.34±0.01**	0.23±0.01***	0.22±0.01***
Sum n−3 (mg/g)	0.73±0.04	0.59±0.01***	0.58±0.01***	0.51±0.01***	0.28±0.01***	0.31±0.01***
Calculated n−6 HUFA (%)	26.6±0.2	36.1±0.5***	39.4.0±0.6***	47.5±0.5***	71±1***	68.0±0.6***
Calculated n−3 index (%)	19.4±0.1	15.9±0.2***	15.2±0.2***	13.3±0.3***	7.7±0.1***	7.8±0.2***

The n−3 index: EPA+ DHA content of erythrocytes expressed as a percent of total fatty acids in RBCs. Sum n−3 and sum n−6 include additional fatty acids not indicated in the table.

Data are presented as mean ± SEM (n = 5). Asterisk(s) indicates significant different from WD-FO.

* p<0.05,

** p<0.01,

*** p<0.005.

Abbreviations: AA, arachidonic acid; DHA, docosahexaenoic acid, EPA, eicosapentaenoic acid, FA, fatty acids; HUFA, highly unsaturated fatty acids (HUFA, ≥20 carbons and ≥3 carbon-carbon double bonds), LA, linoleic acid; MUFA, monounsaturated fatty acids; RBCs, red blood cells; SFA, saturated fatty acids.

### Replacing Fish Oil with Vegetable Oils in Fish Feed Reduces the Levels of PCBs and DDTs in Atlantic Salmon Fillets and Accumulation of these in Adipose Tissue from Mice Consuming the Salmon Fillets

**Figure 2:**
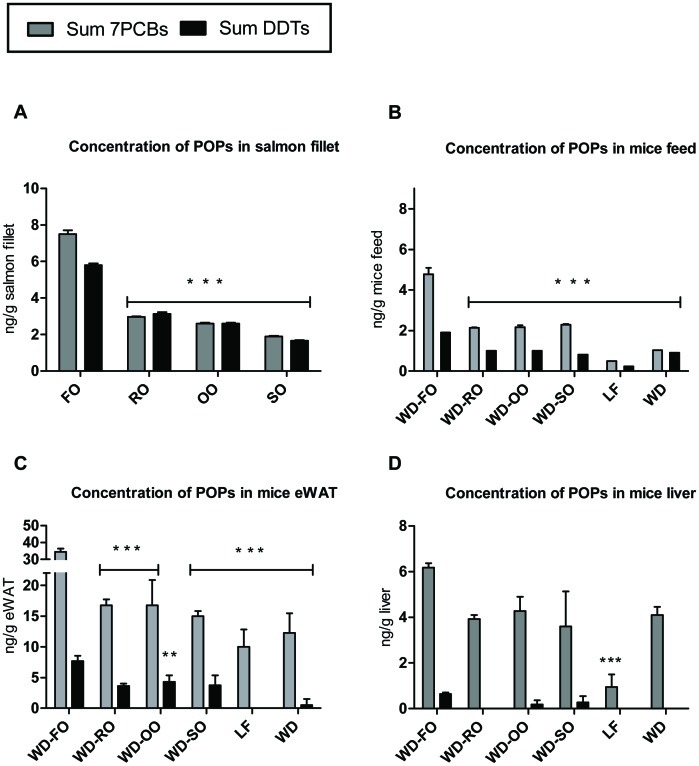
Composition of salmon aqua feed changes accumulation of POPs in mice consuming the salmon fillets. Fish oil (FO) in aqua feed was partly replaced with rapeseed oil (RO), olive oil (OO) or soy bean oil (SO) and fed Atlantic salmon. The salmon fillets were used in Western diets (WDs) fed male C57BL/6J mice (n = 8/diet) for 10 weeks. Concentrations of 7PCBs and DDTs were measured in (A) Atlantic salmon fillets (B) the WDs containing the fillets and (C) epididymal white adipose tissue (eWAT) and (D) liver collected from mice consuming the WDs and a low fat reference diet. Data represent mean of duplicate measurements in A and B. Tissues from two animals were pooled to achieve sufficient material for POP analyzes, and data in C and D thus represent mean+SEM (n = 4). Asterisk(s) indicates significant different from WD-FO.

When FO was replaced with VO in salmon feed, the levels of PCBs and DDTs were reduced by more than 50% in the salmon fillets (Fig. 2A). PCBs and DDTs are lipid soluble contaminants that accumulate in adipose tissue in rats and mice [32], [33]. Thus, the contaminant levels in adipose tissue, but not in liver of the mice mirrored the levels in the salmon fillets and the mouse diets (Fig. 2). Together, changes in the salmon feed were translated into altered fatty acid composition in RBCs and levels of POPs in adipose tissue in mice consuming the salmon fillets.

### The Nutrient- and Contaminant Changes in Salmon Fillets do not Influence Obesity Development in Mice

We have previously demonstrated that chronic consumption of commercially available Atlantic salmon with a high level of POPs caused obesity in obesity prone C57BL/6J mice [33]. When the levels of POPs in the salmon were reduced, obesity development in the mice were slightly, but significantly, decreased. Here we demonstrate that although the accumulation of both PCBs and DDTs in adipose tissue was significantly lower in mice fed the various WD-VOs than in mice fed WD-FO, (Fig. 2C) body weight gain (Table 4) and adipose tissue masses (Table 5) were comparable. As expected, mice fed the LF diet, consumed significantly less energy and gained less weight than WD-FO fed mice. However, energy intake and apparent fat digestibility in the WD-VOs fed mice were comparable to those of mice fed the WD-FO (Table 4). Thus, neither reduced levels of PCBs and DDTs nor lower n−3/n−6 ratio in the WD-VOs had any effect on the body weight or obesity development (Table 4 and 5).

**Table 4:** Body weight, energy intake, energy efficiency and apparent fat digestibility in mice fed the different diets for 10 weeks.

	WD-FO	WD-RO	WD-OO	WD-SO	LF	WD
Initial BW (g)	25.4±0.5	25.3±0.4	25.4±0.5	25.5±0.4	25.4±0.6	25.4±0.5
BW gain (g)	18.6±0.6	16.7±0.9	17.0±1.0	18.5±1.1	8.2±0.7***	15.3±0.7*
Total energy intake (kJ)	4125±140	4016±127	4113±231	4032±195	3423±133*	3840±104
Energy efficiency (g/MJ)	4.5±0.2	4.2±0.3	4.2±0.4	4.6±0.2	2.4±0.2***	4.0±0.2
Fat digestibility (%)	97.7±0.3	98.1±0.3	98.5±0.1	98.6±0.1	96.7±0.1	96.2±0.6**

Data are presented as means ± SEM (n = 8). Asterisk(s) indicates significant different from WD-FO.

* p<0.05,

** p<0.01,

*** p<0.005.

Abbreviations: BW, body weight.

**Table 5:** Organ weights (g) in the mice fed the experimental diets for 10 weeks.

	WD-FO	WD-RO	WD-OO	WD-SO	LF	WD
Liver	1.35±0.06	1.33±0.03	1.54±0.10	1.59±0.14	1.11±0.04	1.47±0.05
eWAT	1.98±0.05	1.97±0.08	1.87±0.16	1.96±0.08	0.70±0.08*	1.79±0.08
iWAT	0.70±0.02	0.68±0.05	0.65±0.09	0.71±0.05	0.25±0.03*	0.63±0.05
iBAT	0.102±0.006	0.085±0.006	0.089±0.008	0.100±0.006	0.080±0.007	0.084±0.005
Muscle (Tibialis)	0.091±0.004	0.081±0.004	0.097±0.006	0.092±0.007	0.089±0.005	0.082±0.003

Data are presented as means ± SEM (n = 8). Asterisk(s) indicates significant different from WD-FO.

* p<0.05,

** p<0.01,

*** p<0.005.

Abbreviations; eWAT, epididymal white adipose tissue; iBAT, interscapular brown adipose tissue; iWAT, inguinal white adipose tissue.

### The Fatty Acid Composition in Salmon Fillets Influences Development of Insulin Resistance and Hepatic Lipid Accumulation in Mice

Given the earlier findings that POPs appear to exacerbate insulin resistance [32], [33], whereas n−3 PUFAs have been demonstrated to attenuate development of diet-induced insulin resistance [9], [11]–[15], we measured fasting levels of glucose and insulin, and performed glucose and insulin tolerance tests.

**Figure 3:**
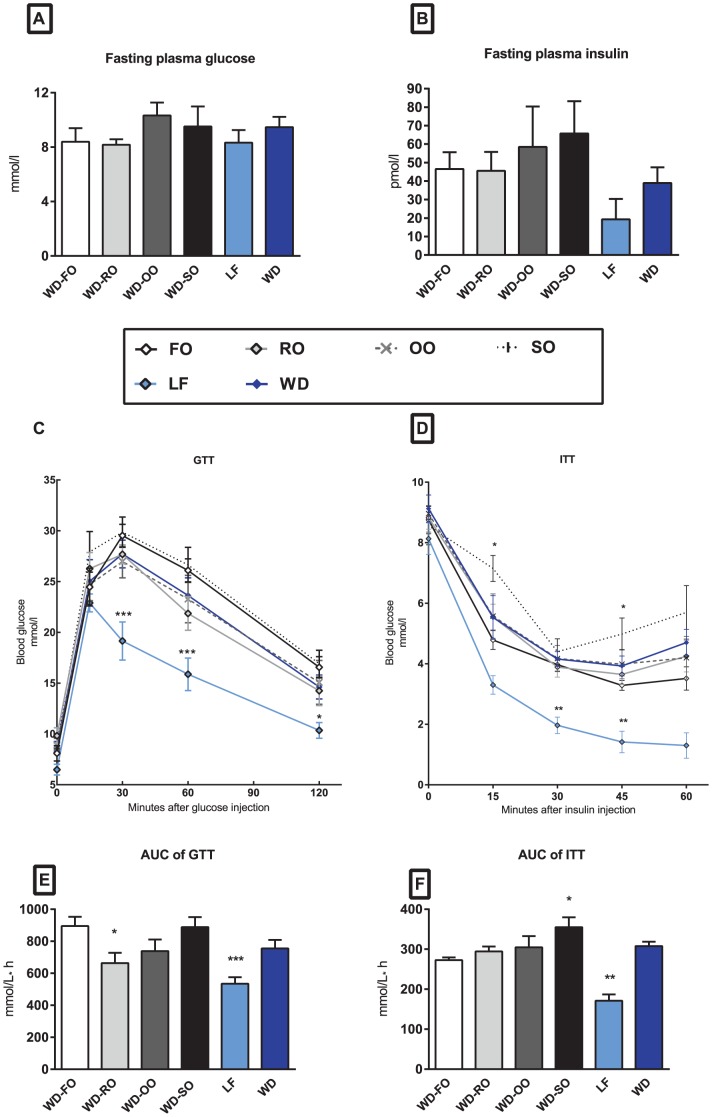
Fatty acid composition in salmon fillets influences development of insulin resistance in mice. Male C57BL/6J mice (n = 8/diet) were fed WD-FO, WD-RO, WD-OO and WD-SO for 10 weeks. As references, two groups of mice received regular WD or LF diet. Plasma glucose (A) and insulin (B) were measured after overnight fasting. An intraperitoneal glucose tolerance test (GTT) was performed after 7 weeks of feeding (C) and an intraperitoneal insulin tolerance test (ITT) was performed after 8 weeks of feeding (D). Area under the curve (AUC) was calculated from the glucose tolerance (baseline was set to fasted blood glucose levels) (E) and insulin tolerance test (F). Data are presented as means+SEM (n = 8). *represents significant different from WD-FO (P<0.05). **represents significant different from WD-FO (P<0.01). ***represents significant different from WD-FO (P<0.005).

Overnight fasting plasma glucose levels were comparable in all groups (Fig. 3A). Compared with intake of WD-FO, intake of WD-VOs did not affect plasma insulin levels (Fig. 3B). The glucose tolerance in mice fed the WD-FO was comparable to the glucose tolerance in mice fed the standard WD, but lower than in mice fed the LF diet (Fig. 3C and E). WD-RO had a significantly decreased incremental area under the curve (AUC) compared with WD-FO (Fig. 3E) indicating an improved glucose tolerance. During the insulin tolerance test, AUC in WD-RO fed mice was not significantly lower than in mice fed WD-SO, but significantly lower (p<0.05) than in WD-FO fed mice (Fig. 3D). Mice receiving WD-SO had significantly higher blood glucose levels at 15 and 45 min after insulin injection than mice fed the WD-FO (Fig. 3D). LF fed mice had lower plasma glucose 30 and 45 min after injection of glucose (Fig. 3D). The difference was confirmed by a repeated-measures ANOVA test during the ITT revealing that the glucose response over time was significantly higher after in WD-SO fed mice as compared to WD-FO fed mice (p<0.05), whereas the glucose response over time in LF fed mice was significantly lower (p<0.01). Furthermore, mice receiving WD-SO had significantly larger AUC during ITT than mice fed the WD-FO (Fig. 3F). These results suggest that insulin sensitivity in mice fed WD-SO was decreased.

**Figure 4:**
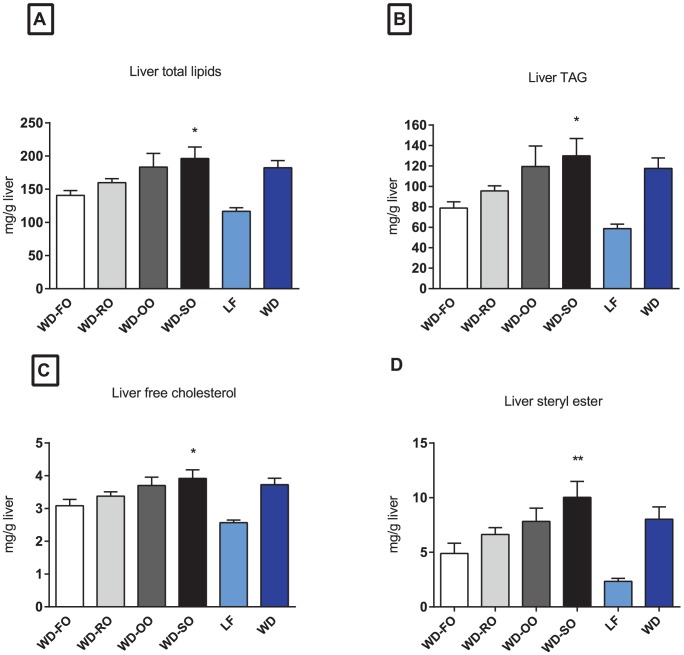
Fatty acid composition in salmon fillets influences development of hepatic lipid accumulation in mice. Male C57BL/6J mice (n = 8/diet) were fed WD-FO, WD-RO, WD-OO and WD-SO for 10 weeks. As references, two groups of mice received standard WD or LF diet. Lipids were extracted from liver and (A) total lipids; (B) triacylglycerol (TAG); (C) free cholesterol, and (D) steryl ester (SE) were quantified. Data are presented as means+SEM (n = 8). Asterisk(s) indicates significant different from WD-FO.

The reduced insulin sensitivity in WD-SO fed mice was not associated with increased adipose tissue mass. However, as not only increased adipose tissue mass, but also ectopic deposition of lipids have been suggested to contribute to the development of insulin resistance [11], [36]–[38] we quantified lipids in liver and the tibialis anterior muscle. We did not observe any significant changes in muscle lipid content (not shown). Hepatic levels of PL and free fatty acids were similar in all groups but mice fed the WD-SO had significantly higher levels of total lipids, free cholesterol, steryl ester (SE) and triacylglycerol (TAG) in the liver than mice fed the WD-FO (Fig. 4). A similar tendency was seen in mice fed the WD-OO, but due to large individual differences this did not reach statistical significance. Given the ability of n−3 PUFAs to protect against diet-induced accumulation of fat in liver [39], [40], and the indication of increased accumulation of lipids with increasing amount of n−6 in the diet, our data suggest that fatty acid composition in the salmon fillets appears to play a more important role than the levels of 7 PCBs and DDTs.

## Discussion

The acceptance of Atlantic salmon as part of a healthy diet has to a large extent been based on its high content of marine n−3 PUFAs. However, the traditional use of fish-derived raw materials such as fish meal and FO in aquaculture is not sustainable due to the pressure on wild fish stocks. Thus, VOs are presently recognized as suitable alternatives in aqua feed [20]. We recently demonstrated that a net production of marine proteins, but not marine n−3 PUFAs, is achievable when 70% of the marine proteins and 80% of the marine oils are replaced by vegetable alternatives [22]. Here we demonstrate that replacing FO with RO, OO or SO, decreased the n−3/n−6 ratio in salmon fillets by 79, 76 and 92%, respectively, with a concomitant reduction in marine n−3 PUFAs in RBCs collected from mice fed WDs containing the salmon fillets. The omega-3 PUFA content in RBC membranes reflects the levels in cardiac membranes, and an omega-3 index below 4% is considered a risk factor for cardiovascular disease [2]. The omega-3 index was lower in mice fed the WD-VOs, WD-SO in particular, than in mice fed the WD-FO, however, the omega-3 index remained above 8% in all mice fed WDs containing salmon. Thus, the cardio-protective effects of salmon ingestion appeared to be maintained in diets based on VO fed salmon.

The ability of marine n−3 PUFAs to protect against development of obesity in rodents is well documented [5]–[10], whereas n−6 PUFAs, LA in particular, have been associated with increased weight gain [10], [41]–[44]. We recently demonstrated that elevation of dietary LA from 1 to 8en% increased endocannabinoid tone and obesity development in mice, an effect that was prevented by adding 1% EPA and DHA to the 8e% LA diets [10]. Furthermore, excessive dietary LA from SO in salmon feed increased the endocannabinoid tone, both in Atlantic salmon and in mice fed high fat diets containing the salmon fillets. The increased endocannabinoid tone in the mice was accompanied by increased weight gain [24]. Here, the WD-SO contained more n−6 PUFAs, mainly LA, than the WD-FO and the other WD-VOs. Thus, the calculated n−6 HUFA content in WD-SO fed mice was significantly higher than in WD-FO and the other WD-VO fed mice. However, obesity development and weight gain were not influenced by replacing FO with VO in the present study. Compared to WD-FO fed mice, glucose tolerance was comparable in mice fed WD-OO and WD-SO, and actually improved in WD-RO fed mice. Despite similar body weight, adipose tissue mass and glucose tolerance, WD-SO fed mice became more insulin resistant and accumulated more lipids in the liver than WD-FO fed mice. Thus, in this study insulin resistance was accompanied by ectopic fat accumulation rather than obesity. Although a causal relationship is not fully established, hepatic insulin resistance is frequently accompanied by excessive deposition of fat in the liver [37]. The sequence of events leading to insulin resistance is not elucidated, but it was recently demonstrated that development of diet-induced hepatic insulin resistance precedes development of peripheral insulin resistance [45]. The finding that WD-SO fed mice had higher accumulation of hepatic TAG and were more insulin resistant than WD-FO fed mice might thus be related to the fatty acid composition of the diets. A number of studies have demonstrated the ability of marine n−3 PUFAs to prevent diet-induced accumulation of hepatic TAG [2], [46], [47]. Of note, the levels of the marine omega-3 fatty acids, EPA and DHA, were comparable in all WD-VOs. However, the level of LA was higher in WD-SO than in WD-OO and WD-RO. This might be of great importance for the omega-3 index, as the conversion of LA to arachidonic acid (AA), allows competition between AA and the n−3 PUFAs, EPA and DHA, for incorporation into membrane phospholipids (PL) [10]. In the study by Alvheim et al, 100% replacement of FO with SO in salmon feed led to increased LA and decreased EPA and DHA levels in salmon liver and fillet [24]. The mice fed SO salmon had higher AA and lower EPA and DHA levels in the PLs. Glucose tolerance and insulin resistance were not measured in the study by Alvheim et al., but the lower levels of EPA and DHA were accompanied with a 2.5-fold increased accumulation of hepatic TAG in mice fed SO salmon. Although a cause relationship is not demonstrated, it is possible that the high levels of n−6 PUFAs, LA in particular, in the WD-SO attenuated the ability of marine n−3 PUFA to reduce hepatic TAG, and thereby contributed to the augmented insulin resistance in WD-SO fed mice.

In the study by Alvheim et al., the Atlantic salmon were fed refined and purified FO and SO. However, the oils commonly used in the aquaculture industry contain POPs, and the use of vegetable oils in aqua feed will introduce different POPs in addition to changing the nutrient composition of salmon fillet. This is an important aspect, as the POPs present in commercially available farmed salmon are transferred to mice consuming the salmon, and these POPs may contribute to development of obesity and insulin resistance [32], [33]. Chronic long term consumption of commercially available Atlantic salmon with current levels of POPs caused obesity and insulin resistance in mice [33], and the accumulation of POPs in adipose tissue was similar to levels in mice fed the WD-FO. When the levels of POPs in the salmon were reduced, obesity development and insulin resistance were slightly, but significantly decreased [33]. In this study, however, neither obesity nor insulin resistance correlated with accumulation of POPs in adipose tissue. Since obesity in general is often associated with insulin resistance, we cannot exclude the possibility that insulin resistance in the earlier studies [32], [33], at least in part, simply reflected the degree of obesity. Laboratory rodents are normally fed a diet where casein is used as the protein source. In a previous salmon study, we replaced 100% of the casein with salmon proteins [33]. Mice receiving salmon as the sole protein source gained significantly more adipose tissue mass than mice receiving casein. The increased adiposity was accompanied with increased digestibility of fat. Thus, in order to limit the difference in fat digestibility, we exchanged only 50% of the casein with proteins from salmon in the present study. The difference in fat digestibility between WD and WD-FO was still significant with higher digestibility in the WD-FO. All the WD-VOs had the same fat digestibility as WD-FO, suggesting that the uptake of fat from the different WDs was comparable.

In the present experiment insulin resistance was more pronounced in mice fed the WD-SO than in mice fed WD-FO although the degree of adiposity was similar in all WD groups. Of note, the levels of PCBs and DDTs reported to accumulate in adipose tissue [33] were comparable to the levels found after 10 weeks feeding with WD-FO in the present study, whereas mice fed the WD-SO diet had lower accumulation of PCBs and DDTs than reported for mice fed Atlantic salmon with reduced POP levels [33]. Obviously, the levels of POPs in adipose tissue are not the sole determining factor of insulin resistance. We cannot exclude the possibility that POPs contributed to the development of insulin resistance, but our results suggest that other factors, such as fatty acid composition in the diets, are of importance.

In view of the limited access to marine fish meal and fish oil for the rapidly growing aquaculture industry, a global contributor to food security, the use of alternative feed ingredients is inevitable. Exchanging FO with VO in aquatic feed reduced the accumulation of POPs in adipose tissue by more than 50% in mice consuming the salmon. Importantly, in relation to replacement of fish oil with vegetable oil in future feed for salmon farming, our data clearly demonstrate that VOs have markedly different spillover effects on metabolism in mice, and that the content of LA may be a matter of concern. This raises the important question as to whether similar effects will be observed in humans consuming Atlantic salmon fed VOs, SO in particular, and in this context our study suggests that RO and/or OO represent a better choice than SO to replace FO.
